# Factors associated with sarcopenia screened by finger-circle test among middle-aged and older adults: a population-based multisite cross-sectional survey in Japan

**DOI:** 10.1186/s12889-021-10844-3

**Published:** 2021-04-26

**Authors:** Daiki Watanabe, Tsukasa Yoshida, Takashi Nakagata, Naomi Sawada, Yosuke Yamada, Kayo Kurotani, Kenji Tanaka, Megumi Okabayashi, Hidekazu Shimada, Hidemi Takimoto, Nobuo Nishi, Keiichi Abe, Motohiko Miyachi

**Affiliations:** 1 National Institute of Health and Nutrition, National Institutes of Biomedical Innovation, Health and Nutrition, 1-23-1 Toyama, Shinjuku-ku, Tokyo, 162-8636 Japan; 2 Institute for Active Health, Kyoto University of Advanced Science, 1-1 Nanjo Otani, Sogabe-cho, Kameoka-city, Kyoto, 621-8555 Japan; 3 Department of Health and Welfare, Settsu City Local Government, 1-1-1 Mishima, Settsu-city, Osaka, 566-8555 Japan; 4 Present Address: Department of Developing Next Generation, Settsu City Local Government, 1-1-1 Mishima, Settsu-city, Osaka, 566-8555 Japan; 5 Faculty of Life and Environmental Sciences, Showa Women’s University, 1-7-57 Taishido, Setagaya-ku, Tokyo, 154-8533 Japan; 6 Department of Health, Hannan City Local Government, 35-1 Ozaki-cho, Hannan-city, Osaka, 599-0201 Japan; 7 Department of Public Health and Medical Affairs, Osaka Prefectural Government, 2-1-22 Otemae, Chuo-ku, Osaka-city, Osaka, 540-8570 Japan; 8 National Institutes of Biomedical Innovation, Health and Nutrition, 7-6-8 Saito-Asagi, Ibaraki-city, Osaka, 567-0085 Japan

**Keywords:** Sarcopenia, Random cluster sampling, Middle-aged and older adults, Finger-circle (yubi-wakka) test, Multisite cross-sectional survey

## Abstract

**Background:**

Previous epidemiological studies have demonstrated the prevalence and relationship of various factors associated with sarcopenia in older adults; however, few have examined the status of sarcopenia in middle-aged adults. In this study, we aimed to, 1) evaluate the validity of the finger-circle test, which is potentially a useful screening tool for sarcopenia, and 2) determine the prevalence and factors associated with sarcopenia in middle-aged and older adults.

**Methods:**

We conducted face-to-face surveys of 525 adults, who were aged 40–91 years and resided in Settsu City, Osaka Prefecture, Japan to evaluate the validity of finger-circle test. The finger-circle test evaluated calf circumference by referring to an illustration printed on the survey form. The area under the receiver operating characteristic curves (AUROC) was plotted to evaluate the validity of the finger-circle test for screening sarcopenia and compared to that evaluated by skeletal muscle mass index (SMI) measured using bioimpedance. We also conducted multisite population-based cross-sectional anonymous mail surveys of 9337 adults, who were aged 40–97 years and resided in Settsu and Hannan Cities, Osaka Prefecture, Japan. Participants were selected through stratified random sampling by sex and age in the elementary school zones of their respective cities. We performed multiple logistic regression analysis to explore associations between characteristics and prevalence of sarcopenia.

**Results:**

Sarcopenia, defined by SMI, was moderately predicted by a finger-circle test response showing that the subject’s calf was smaller than their finger-circle (AUROC: 0.729, < 65 years; 0.653, ≥65 years); such subjects were considered to have sarcopenia. In mail surveys, prevalence of sarcopenia screened by finger-circle test was higher in older subjects (approximately 16%) than in middle-aged subjects (approximately 8–9%). In a multiple regression model, the factors associated with sarcopenia were age, body mass index, smoking status, self-reported health, and number of meals in all the participants.

**Conclusions:**

Sarcopenia, screened by the finger-circle test, was present not only among older adults but also among middle-aged adults. These results may provide useful indications for developing public health programs, not only for the prevention, but especially for the management of sarcopenia.

**Trial registration:**

UMIN000036880, registered prospectively May 29, 2019, https://upload.umin.ac.jp/cgi-open-bin/ctr_e/ctr_view.cgi?recptno=R000042027

**Supplementary Information:**

The online version contains supplementary material available at 10.1186/s12889-021-10844-3.

## Background

Sarcopenia, a syndrome characterized by reduced physical function associated with the loss of skeletal muscle mass and strength [1–4], is associated with adverse health outcomes, such as increased mortality [5, 6] and disabilities [7], among community-dwelling adults aged ≥65 years. In 2016, sarcopenia was recognized as a disease in the International Classification of Diseases-10 [8]. In addition, sarcopenia is reportedly associated with the risk of mortality in patients with nonmetastatic breast cancer [9]. Therefore, reducing the number of patients with sarcopenia is important from a public health perspective to extend healthy lifespans, and from a clinical perspective to reduce healthcare and nursing care expenses [10]. Determining the prevalence of sarcopenia and its associated factors is essential for establishing an effective sarcopenia prevention program.

Calf circumference is strongly associated with appendicular skeletal muscle mass (ALM) in middle-aged and older adults [11–13]. The Asian Working Group for Sarcopenia (AWGS) 2019 Consensus developed a set of guidelines for diagnosing and treating sarcopenia in Asians and proposed the calf circumference as a muscle mass diagnosing tool for sarcopenia [2]. Subsequently, the need for a simpler approach than the calf circumference led to the development of the finger-circle (yubi-wakka) test, which can be conducted by the subjects using their own fingers and does not require any special devices [14, 15]. This test is reportedly associated with psoas muscle mass [15], sarcopenia [14], and the risk of mortality [14] among older adults. Therefore, the finger-circle test could serve as an effective sarcopenia screening tool.

Studies among community-dwelling older adults have reported that factors such as age [16–20], sex [16, 19, 21], body mass index (BMI) [16–18, 20], smoking status [22], and chronic conditions [16, 18, 19, 23] are associated with the prevalence of sarcopenia. However, since these studies were on older adults, little is known about the developmental status of sarcopenia in middle-aged adults. In addition, due to the low statistical power of small sample sizes, studies that randomly sample large populations to explore factors associated with sarcopenia are needed. To the best of our knowledge, the prevalence and factors associated with sarcopenia in middle-aged and older Japanese adults have not yet been determined. Life-style intervention programs can reduce the progression of functional decline among older adults with moderate physical disorder but not those with severe physical disorder [24]. Therefore, identifying these associations is essential for early detection and prevention of sarcopenia in those aged 30 years and above, when the muscle mass and strength begin to decline [25]. In this study, we aimed to 1) evaluate the validity of the finger-circle test for screening sarcopenia using face-to-face surveys and 2) determine the prevalence and factors associated with sarcopenia screened by finger-circle test among randomly selected middle-aged and older adults, based on the demographic ratios of sex and age group in the general populations of Settsu and Hannan Cities, Osaka Prefecture, Japan.

## Methods

### Study population

**Fig. 1:**
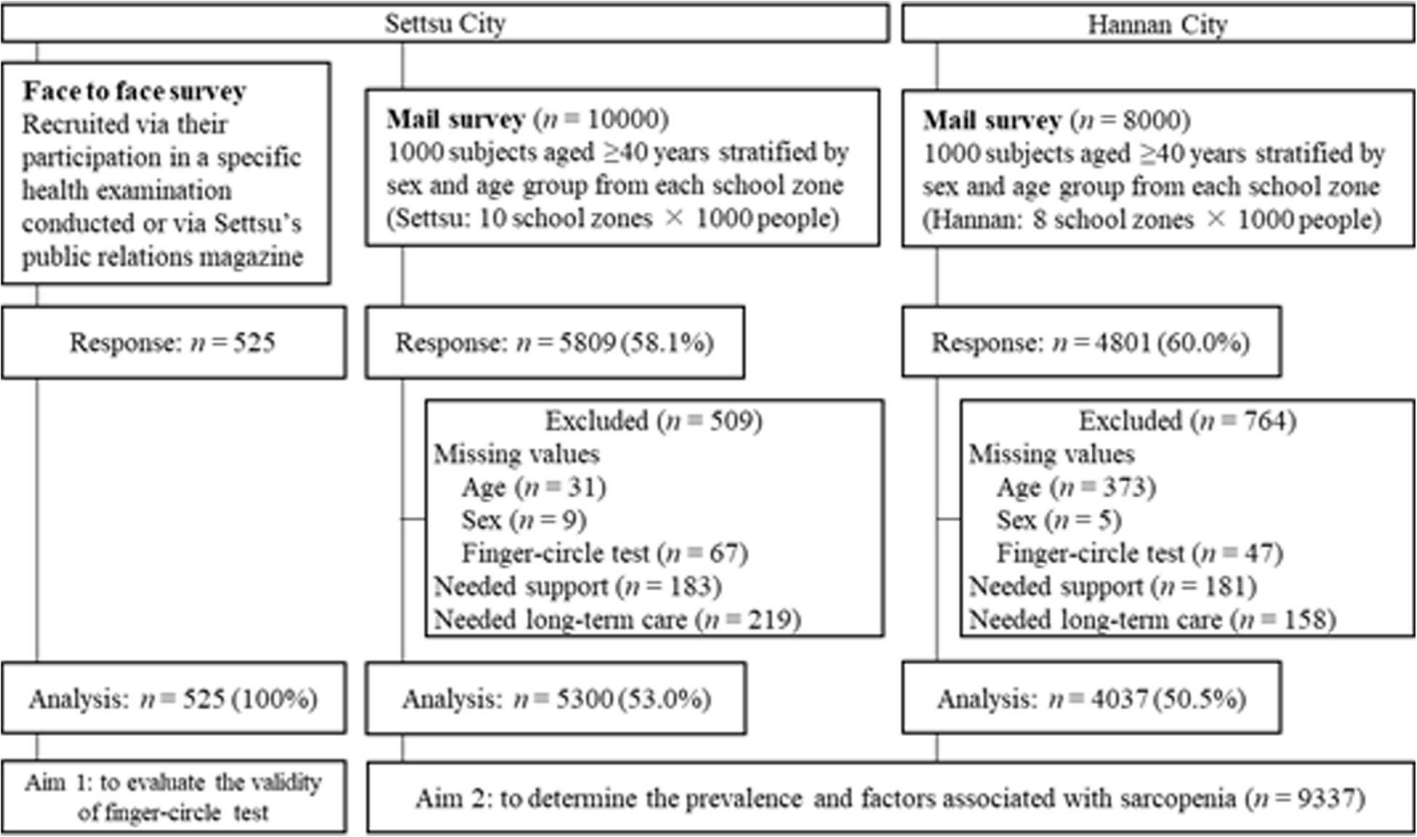
Flow chart of the study participants

From July 1 to July 9, 2019, we conducted face-to-face surveys of 525 men and women, who were aged ≥40 years and resided in Settsu City. These participants were recruited either when they participated in a specific health examination conducted at the Settsu Health Center or via Settsu’s public relations magazine (Fig. 1). We assessed the validity of the finger-circle test for assessing sarcopenia by comparing the test data obtained from participants aged 40–91 years with their body composition-based assessment data.

The present study is a multisite population-based cross-sectional anonymous mail survey of men and women, who were aged ≥40 years and resided in the cities of Settsu and Hannan in Osaka Prefecture, Japan. Settsu is a municipality in Mishima District (population: 85007; area: 14.87 km^2^) in northern Osaka Prefecture [26], while Hannan is a municipality in Sennan District in southern Osaka Prefecture (population: 54276; area: 36.17 km^2^) [26]. Settsu and Hannan have 10 and 8 elementary school zones, respectively. To obtain a representative sample of the general population, we randomly selected 1000 subjects, who were aged ≥40 years and stratified by sex and age from each school zone (Fig. 1). Survey forms and envelopes were color-coded by school zone to identify each school zone respondent when their survey forms were returned. The Settsu mail survey was conducted from February 22, 2019 to March 5, 2019. Although 4502 survey forms were returned by March 7, 2019, additional 1307 forms that were returned after that date were also included in the analysis (response rate: 58.1%). The Hannan mail survey was conducted from January 20, 2020 to February 7, 2020. Survey forms were included only if they arrived by February 13, 2020 (response rate: 60.0%). For both surveys, we sent a reminder postcard 1 week prior to the end of the survey period.

Of these participants, we excluded those with missing data on finger-circle test, age, or sex, and those who had self-reported “needed long-term care” or “needed support”. Ultimately, we included 9337 participants to this study.

### Survey content

In the Settsu and Hannan mail surveys, we collected the following basic characteristics: age (years); sex (male or female); certification of needed support or long-term care (none, needed support level 1–2, needed long-term care level 1–5); height (centimeters); body weight (kilograms); drinking status (“Do you drink alcohol?”: almost daily, sometimes, almost never, never); smoking status (“Do you smoke?”: almost daily; sometimes; used to, but quit; never); living status (“Do you live alone?”: yes, no); socioeconomic status (“Economically, how does your life feel currently?”: hard, somewhat hard, somewhat easy, easy); self-reported health (“How healthy do you normally feel?”: very healthy, somewhat healthy, not very healthy, unhealthy); self-reported physical fitness (“How confident are you in your current physical fitness?”: extremely confident, somewhat confident, slightly anxious, very anxious); exercise habits (“Do you go walking or engage in other exercise at least once per week?”: yes, no); mastication function (“Is it more difficult for you to eat hard foods now than it was six months ago?”: yes, no); number of meals (“How many meals do you eat per day (excluding snacks)?”: [number of meals]); sleep status (“In the past month, have you been getting enough rest from sleep?”: yes, somewhat, not very much, not at all); short-term cognitive ability (“Can you remember what happened 5 minutes ago?”: yes, no); and gait speed (“Do you feel like your gait speed is slower than it used to be?”: yes, no). BMI was calculated as body weight (kg) divided by the square of height (m).

### Definition of sarcopenia

In the face-to-face survey, height was measured in 0.1 cm increments using a stadiometer with the participants’ shoes removed. Body composition was assessed via an octopolar multi-frequency bioimpedance device (MC-780A, TANITA Corp., Tokyo, Japan) which was validated against Dual-energy X-ray absorptiometry (DEXA) [27], with participants wearing as light clothing as possible. It was reported that sarcopenia defined only by low muscle mass was similar to sarcopenia defined according to the criteria of The European Working Group on Sarcopenia in Older People (EWGSOP) [28] or AWGS [29] (low muscle mass, low muscle strength, and low physical performance); hence, sarcopenia was defined by a skeletal muscle mass index (SMI) (evaluated by the bioimpedance method) of < 7.0 kg/m^2^ in men and < 5.7 kg/m^2^ in women, in accordance with the AWGS 2019 Consensus, in our study [2]. SMI was calculated as the ALM (kg) divided by the square of the height (m^2^).

**Fig. 2:**
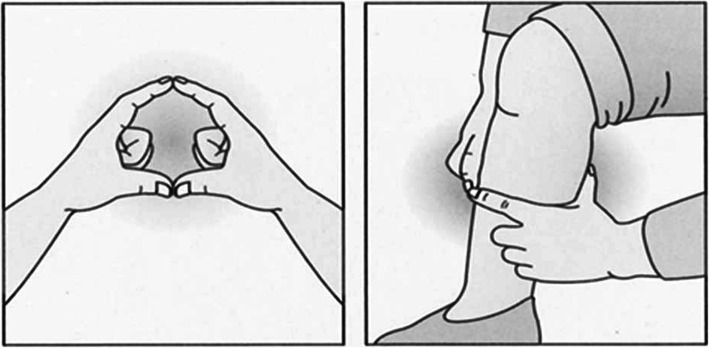
Finger circle (yubi-wakka) test

In the mail survey, sarcopenia was assessed using self-reported finger-circle test data, that was validated in adults aged ≥65 years [14, 15]. The finger-circle (yubi-wakka) test determines whether the circumference of a participant’s finger, formed by their index fingers and thumbs, is smaller than the participant’s maximum non-dominant calf circumference. This test is explained in detail elsewhere [14, 15]. By referring to an illustration printed on the survey form, we asked the participants to conduct the finger-circle test according to the following instructions (Fig. 2): “Put your two index fingers and thumbs together to make a circle around your calf. Compare the size of this finger-circle to the thickness (circumference) of the thickest part of your calf (calf is smaller [smaller], calf and finger-circle are about the same size [same size], or calf is bigger [bigger]).” A prospective cohort study revealed an association between the finger-circle test outcomes and sarcopenia and mortality risks in adults, who were aged ≥65 years [14].

### Statistical analysis

Settsu and Hannan mail survey data were analyzed separately. Continuous and ordinal participant characteristics were classified for: age (40–49, 50–59, 60–69, 70–79, and 80 years); BMI (< 18.5, 18.0–24.9, 25.0–29.9, and ≥ 30.0 kg/m^2^); alcohol consumption (“almost daily,” “sometimes,” or “almost never” = alcohol drinkers and “never” = non-drinkers); smoking (“almost daily” or “sometimes” = current smokers, “used to, but quit” = past smokers, and “never” = never-smokers); socioeconomic status (“easy” or “somewhat easy” = high socioeconomic status, and “somewhat hard” or “hard” = low socioeconomic status); self-reported health (“very healthy” or “somewhat healthy” = good self-reported health, and “not very healthy” or “unhealthy” = poor self-reported health); self-reported physical fitness (“extremely confident” or “somewhat confident” = good self-reported physical fitness, and “slightly anxious” or “very anxious” = poor self-reported physical fitness); number of meals (≥3, 2, and 1 meal); sleep status (“yes” and “somewhat” = good sleep status, while “not very much” and “not at all” = poor sleep status). These variables were classified with reference to covariates used in a previous study [30]. Participant characteristics were described for the variables mentioned above. Continuous variables are shown as means and standard deviations, while categorical variables are shown as numbers and percentages. Missing values for these variables were created from five datasets by multiple imputation by chained equation [31]. All missing values were assumed to be missing at random. In addition, complete cases (cases with no missing values) and cases with missing values were compared by age, sex, and prevalence of sarcopenia screened by finger-circle test.

In the face-to-face survey, height, and body composition data for each group of finger-circle test responses (bigger, same size, smaller) are shown as means and standard deviations. The *p*-values for linear trends were calculated by using the finger-circle test (the exposure variable) as a continuous variable. To examine the validity of the finger-circle test in screening sarcopenia, we calculated the area under the receiver operating characteristic curves (AUROC) for the finger-circle test in relation to sarcopenia defined using SMI. Previous studies verified the validity of the finger-circle test only in adults, who were aged ≥65 years [14, 15]. Therefore, in our analyses, we stratified the participants by age (≥65 or < 65 years) and sex (male or female).

Prevalence of sarcopenia screened by finger-circle test was reported as number of cases and percentages for the participants in the mail survey. To adjust for the distribution ratio of the sample in the mail survey participants to the distribution ratio of Settsu and Hannan populations, we calculated the prevalence of sarcopenia weighted by the distribution ratios according to age (40–49, 50–59, 60–69, 70–79, and ≥ 80 years) and sex (male or female) in each school zone [32]. We used the chi-square test to compare characteristics between the sarcopenia and non-sarcopenia groups.

We used multiple logistic regression analysis to examine factors associated with the prevalence of sarcopenia screened by finger-circle test in the mail survey participants. In our multivariate analysis, the dependent variable was the prevalence of sarcopenia as assessed by the finger-circle test; while the explanatory variables were age, sex, BMI, alcohol consumption, smoking status, living status, socioeconomic status, self-reported health, self-reported physical fitness, exercise habits, mastication function, number of meals, sleep status, short-term cognitive ability, and gait speed. We chose this model because a previous study reported that most of the above variables are associated with the prevalence of sarcopenia [33]. The results of these analysis were reported as odds ratios (OR) and 95% confidence intervals (CI). For each variable, the following reference groups were used to calculate the OR: age 40–49 years, male sex, BMI 18.5–24.9 kg/m^2^, non-drinker, never-smoker, living together, high socioeconomic status, good self-reported health, good self-reported physical fitness, “yes” to exercise habits, good mastication function, ≥3 meals, good sleep status, good short-term cognitive ability, and normal gait speed. We also conducted a similar analysis including all the participants. To conduct the sensitivity analysis for assessing the robustness of the above results, we conducted a similar analysis using the complete cases dataset [34].

In the statistical analyses, *p*-values < 0.05 were considered significant. All analyses were performed using the JMP Pro, version 13.2 (SAS Institute, Inc., Cary, NC, USA) and/or R software 3.4.3 (R Development Core Team, Vienna, Austria).

### Ethical considerations

Regarding the Settsu and Hannan mail surveys, we filed an application for an ethical review with the National Institutes of Biomedical Innovation, Health and Nutrition Institutional Review Board (Number: kenei 89, December 3, 2018). An ethical review was deemed unnecessary for the following reasons: 1) no one other than the supervisors at Settsu and Hannan had access to the residents’ information; and 2) the mail surveys were anonymous and contained no information that could be used to identify the individuals. When we sent out the survey forms, we included a document that explained the following: the objective of the study, our methods for selecting the participants, that the returning of the survey form constituted consent to participate in the study, and that the survey forms were color-coded by school zone. The Settsu City face-to-face survey was approved by the Research Ethics Committee of the National Institutes of Biomedical Innovation, Health and Nutrition (Ikikenhatsu-178-1) and has been registered with the University Hospital Medical Information Network in Japan clinical trial registration system (UMIN000036880). Study procedures as well as the risks associated with participating were explained and written informed consent was obtained from all the participants. Moreover, all the study procedures were performed in accordance with relevant guidelines/regulations.

## Results

### Participant characteristics

Table 1 shows the participants’ characteristics for the mail and face-to-face surveys. In the mail survey, we did not observe any major differences in characteristics between Settsu and Hannan participants. The face-to-face survey participants, in whom we measured the body composition, were older and comprised of a higher percentage of women than the mail survey participants. Participants with missing values were largely older adults but did not differ from those with complete cases in terms of sex (see Table S1 in Additional file 1).

**Table 1:** Participant characteristics in mail and face-to-face surveys

	Mail survey ^a^		Face-to-face
	Settsu City (*n* = 5300)	Hannan City (*n* = 4037)	Settsu City (*n* = 525)
Age [years] ^b^	62.8 (12.5)	63.5 (12.2)	70.6 (9.9)
Women [*n* (%)] ^c^	2496 (47.1)	1965 (48.7)	436 (83.0)
BMI [kg/m^2^] ^b^	23.2 (3.6)	23.1 (3.8)	22.5 (3.2)
Alcohol drinker [*n* (%)] ^c^	3844 (72.5)	2836 (70.3)	312 (59.4)
Current smoker [*n* (%)] ^c^	1045 (19.7)	581 (14.4)	18 (3.4)
Living alone [*n* (%)] ^c^	694 (13.1)	477 (11.8)	131 (25.0)
High socioeconomic status [*n* (%)] ^c^	2819 (53.2)	2386 (59.1)	385 (73.3)
Poor self-reported health [*n* (%)] ^c^	1027 (19.4)	595 (14.7)	85 (16.2)
Poor self-reported PF [*n* (%)] ^c^	2553 (48.2)	1707 (42.3)	256 (48.8)
Exercise habits per week [*n* (%)] ^c^	2813 (53.1)	2265 (56.1)	129 (24.6)
Poor mastication [*n* (%)] ^c^	1035 (19.5)	653 (16.2)	79 (15.0)
Number of meals ≥3 times [*n* (%)] ^c^	4548 (85.8)	3588 (88.9)	493 (93.9)
Poor sleep status [*n* (%)] ^c^	1428 (26.9)	912 (22.6)	111 (21.1)
Poor short cognitive ability [*n* (%)] ^c^	446 (8.4)	277 (6.9)	57 (10.9)
Slow gait speed [*n* (%)] ^c^	1840 (34.7)	1294 (32.1)	238 (45.3)

*BMI* body mass index, *PF* physical fitness

^a^Data for participants with missing values in mail survey were imputed using multiple imputation (*n* = [*n* in Settsu City] and [*n* in Hannan City]): BMI (*n* = 208 and 210), alcohol status (*n* = 28 and 8), smoking status y (*n* = 73 and 6), family structure (*n* = 141 and 8), socioeconomic status (*n* = 126 and 143), self-reported health (*n* = 83 and 107), self-reported physical fitness (*n* = 82 and 109), exercise habits (*n* = 91 and 106), mastication (*n* = 67 and 114), number of meals (*n* = 37 and 173), sleep status (*n* = 80 and 109), cognitive status (*n* = 98 and 106), and gait speed (*n* = 93 and 110)

^b^Continuous variables were shown in mean (standard deviation)

^c^Category variables were shown in the number of people (%)

### Validity of the finger-circle test as an assessment of sarcopenia

Table S2 in Additional file 1 shows the relationships of the finger-circle test results with the height and body composition. The finger-circle test demonstrated significant associations with body fat, ALM, SMI, and other body composition elements. Table 2 shows the validity of the finger-circle test for screening sarcopenia. The prevalence of sarcopenia (by SMI in the face-to-face study) was 9.3% (49/525). Finger-circle test response of “calf is smaller than finger-circle” was moderately predictive of the presence of sarcopenia as defined by SMI (AUROC: 0.666 [95% CI: 0.588 to 0.744]). The same relationship was observed when we stratified the participants by age or sex. Therefore, we considered the participants whose finger-circle test response was “calf is smaller than finger-circle” as having sarcopenia.

**Table 2:** Area under the receiver operating characteristic curves for the finger-circle (yubi-wakka) test

	Finger-circle test ^a^			By finger-circle test (smaller) ^c^						
	Bigger	Just fits	Smaller	Sensitivity	Specificity	PPV	NPV	LR+	LR-	AUROC (95% CI)
**Total (*n*** **= 525)**										
Sarcopenia, *n* ^b^	11 (2.1)	21 (4.0)	17 (3.2)	34.7	87.6	22.4	92.9	2.8	0.7	0.666 (0.588 to 0.744)
Non-sarcopenia, *n*	223 (42.5)	194 (37.0)	59 (11.2)							
**Women (*n*** **= 436)**										
Sarcopenia, *n* ^b^	6 (1.4)	14 (3.2)	13 (3.0)	39.4	87.1	20.0	94.6	3.1	0.7	0.691 (0.543 to 0.839)
Non-sarcopenia, *n*	183 (41.9)	168 (38.5)	52 (12.0)							
**Men (*n*** **= 89)**										
Sarcopenia, *n* ^b^	5 (5.6)	7 (7.9)	4 (4.5)	25.0	90.4	36.4	84.6	2.6	0.8	0.641 (0.549 to 0.733)
Non-sarcopenia, *n*	40 (44.9)	26 (29.2)	7 (7.9)							
**< 65 years (*n*** **= 107)**										
Sarcopenia, *n* ^b^	1 (0.9)	1 (0.9)	2 (1.9)	50.0	94.2	25.0	98.0	8.6	0.5	0.729 (0.459 to 0.999)
Non-sarcopenia, *n*	52 (48.6)	45 (42.1)	6 (5.6)							
**≥65 years (*n*** **= 418)**										
Sarcopenia, *n* ^b^	10 (2.4)	20 (4.8)	15 (3.6)	33.3	86.5	22.1	91.4	2.3	0.8	0.653 (0.571 to 0.735)
Non-sarcopenia, *n*	171 (40.9)	149 (35.6)	53 (12.7)							

*AUROC* area under the receiver operating characteristic curves, *CI* confidence interval, *LR+* positive likelihood ratio, *LR−* negative likelihood ratio, *NPV* negative predictive value, *PPV* positive predictive value

^a^Compare the size of the finger-circle to the thickness (circumference) of the thickest part of your calf (calf is smaller [smaller], calf and finger-circle are about the same size [same size], or calf is bigger [bigger])”

^b^Sarcopenia was defined by a skeletal muscle mass index (evaluated by the bioimpedance method) of < 7.0 kg/m^2^ in men and < 5.7 kg/m^2^ in women, in accordance with the AWGS 2019 Consensus

^c^Those whose finger-circle test response was “smaller” were defined as having sarcopenia

### Prevalence and factors associated with sarcopenia

Table 3 shows the prevalence of sarcopenia according to the finger-circle test in the Settsu and Hannan mail surveys. The weighted prevalence of sarcopenia in Settsu and Hannan was 12.9% (95% CI: 12.0 to 13.8) and 12.8% (95% CI: 11.7 to 13.8), respectively. While the prevalence of sarcopenia was higher in older adults (approximately 16%) than in middle-aged adults, sarcopenia was nevertheless present in approximately 8–9% of middle-aged participants (aged 40–59 years). Table 4 shows the comparisons of characteristics between participants with and without sarcopenia according to the finger-circle test. Factors that were associated with the prevalence of sarcopenia in both Settsu and Hannan were age, BMI, smoking status, self-reported health, and mastication function. Table 5 shows the multivariate analysis factors associated with the prevalence of finger-circle test sarcopenia. The ORs for the prevalence of sarcopenia were high with old age, low BMI, smoking, and poor self-reported health in participants from both Settsu and Hannan. In the analysis including all the participants, the prevalence of sarcopenia was additionally related to the number of meals. The same results were obtained when sensitivity analysis was conducted with complete cases (see Table S3 in Additional file 1).

**Table 3:** Age and sex stratified prevalence of sarcopenia screened by finger-circle test in Settsu and Hannan

	Settsu City			Hannan City		
	*n*	Crude	Weighted tabulation	*n*	Crude	Weighted tabulation
Total						
40–49 years	1104	8.7 (7.0 to 10.4)	8.8 (7.1 to 10.5)	701	8.3 (6.2 to 10.3)	8.4 (6.3 to 10.4)
50–59 years	965	8.5 (6.7 to 10.3)	8.5 (6.8 to 10.3)	785	9.3 (7.3 to 11.3)	9.4 (7.4 to 11.4)
60–69 years	1343	12.3 (10.5 to 14.0)	12.1 (10.4 to 13.9)	1088	10.1 (8.3 to 11.9)	10.4 (8.6 to 12.2)
70–79 years	1488	17.6 (15.7 to 19.5)	17.7 (15.8 to 19.6)	1144	16.4 (14.3 to 18.6)	16.5 (14.4 to 18.7)
≥ 80 years	400	21.8 (17.7 to 25.8)	23.7 (19.5 to 27.8)	319	22.9 (18.3 to 27.5)	22.6 (18.0 to 27.2)
Overall	5300	13.1 (12.1 to 14.0)	12.9 (12.0 to 13.8)	4037	12.4 (11.4 to 13.5)	12.8 (11.7 to 13.8)
Men						
40–49 years	646	9.4 (7.2 to 11.7)	9.5 (7.2 to 11.7)	378	9.5 (6.6 to 12.5)	9.9 (6.9 to 12.9)
50–59 years	553	10.5 (7.9 to 13.0)	10.5 (8.0 to 13.1)	413	8.7 (6.0 to 11.4)	9.0 (6.2 to 11.7)
60–69 years	713	13.2 (10.7 to 15.7)	13.1 (10.6 to 15.5)	578	10.7 (8.2 to 13.2)	10.7 (8.2 to 13.2)
70–79 years	717	16.3 (13.6 to 19.0)	16.6 (13.9 to 19.3)	559	16.5 (13.4 to 19.5)	16.4 (13.3 to 19.4)
≥ 80 years	175	26.3 (19.8 to 32.8)	30.5 (23.7 to 37.3)	144	23.6 (16.7 to 30.5)	21.4 (14.7 to 28.1)
Overall	2804	13.4 (12.1 to 14.7)	13.6 (12.3 to 14.9)	2072	12.5 (11.1 to 14.0)	12.6 (11.2 to 14.1)
Women						
40–49 years	458	7.6 (5.2 to 10.1)	7.8 (5.4 to 10.3)	323	6.8 (4.1 to 9.6)	7.2 (4.4 to 10.0)
50–59 years	412	5.8 (3.6 to 8.1)	6.0 (3.7 to 8.3)	372	9.9 (6.9 to 13.0)	10.1 (7.0 to 13.2)
60–69 years	630	11.3 (8.8 to 13.7)	11.0 (8.6 to 13.5)	510	9.4 (6.9 to 11.9)	9.7 (7.1 to 12.3)
70–79 years	771	18.8 (16.0 to 21.6)	19.1 (16.4 to 21.9)	585	16.4 (13.4 to 19.4)	16.5 (13.5 to 19.5)
≥ 80 years	225	18.2 (13.2 to 23.3)	21.4 (16.1 to 26.8)	175	22.3 (16.1 to 28.5)	21.6 (15.5 to 27.7)
Overall	2496	12.7 (11.4 to 14.0)	12.4 (11.1 to 13.7)	1965	12.3 (10.9 to 13.8)	12.6 (11.2 to 14.1)

The values represent the prevalence of sarcopenia (95% confidence intervals). Weighted tabulation was calculated by adjusting the composition ratio of the samples collected from the mail survey to the composition ratio of the population provided by the municipalities of Settsu and Hannan Cities

**Table 4:** Comparison of participant characteristics between sarcopenia and non-sarcopenia groups screened by finger-circle test

	Settsu City		*p*-value	Hannan City		*p*-value
	Non-sarcopenia (*n* = 4608)	Sarcopenia (*n* = 692)		Non-sarcopenia (*n* = 3535)	Sarcopenia (*n* = 502)	
	*n* (%)	*n* (%)		*n* (%)	*n* (%)	
Age [years]						
40–49	1008 (91.3)	96 (8.7)	< 0.001	643 (91.7)	58 (8.3)	< 0.001
50–59	883 (91.5)	82 (8.5)		712 (90.7)	73 (9.3)	
60–69	1178 (87.7)	165 (12.3)		978 (89.9)	110 (10.1)	
70–79	1226 (82.4)	262 (17.6)		956 (83.6)	188 (16.4)	
≥ 80	313 (78.3)	87 (21.8)		246 (77.1)	73 (22.9)	
Women	2180 (47.3)	316 (45.7)	0.419	1723 (48.7)	242 (48.2)	0.823
BMI [kg/m^2^]						
< 18.5	196 (57.0)	148 (43.0)	< 0.001	122 (51.3)	116 (48.7)	< 0.001
18.5–24.9	3031 (86.3)	481 (13.7)		2482 (88.0)	340 (12.0)	
25.0–29.9	1149 (95.3)	57 (4.7)		776 (95.1)	40 (4.9)	
≥ 30	232 (97.5)	6 (2.5)		155 (96.3)	6 (3.7)	
Alcohol drinker	3371 (73.2)	473 (68.4)	0.009	2498 (70.7)	338 (67.3)	0.129
Smoking status						
Never	2295 (87.4)	330 (12.6)	0.036	1973 (88.6)	255 (11.4)	0.049
Past	1426 (87.5)	204 (12.5)		1056 (86.0)	172 (14.0)	
Current	887 (84.9)	158 (15.1)		506 (87.1)	75 (12.9)	
Living alone	601 (13.0)	93 (13.4)	0.774	409 (11.6)	68 (13.5)	0.207
High socioeconomic status	2465 (53.5)	354 (51.2)	0.251	2089 (59.1)	297 (59.2)	0.977
Poor self-reported health	862 (18.7)	165 (23.8)	0.002	493 (13.9)	102 (20.3)	< 0.001
Poor self-reported PF	2197 (47.7)	356 (51.4)	0.065	1489 (42.1)	218 (43.4)	0.580
Exercise habits per	2446 (53.1)	367 (53.0)	0.982	1978 (56.0)	287 (57.2)	0.607
Poor mastication	850 (18.4)	185 (26.7)	< 0.001	556 (15.7)	97 (19.3)	0.045
Number of meals [times]						
1	50 (83.3)	10 (16.7)	0.339	21 (80.8)	5 (19.2)	0.083
2	602 (87.0)	90 (13.0)		364 (86.1)	59 (13.9)	
≥ 3	3956 (87.0)	592 (13.0)		3150 (87.8)	438 (12.2)	
Poor sleep status	1239 (26.9)	189 (27.3)	0.815	802 (22.7)	110 (21.9)	0.697
Poor short cognitive ability	378 (8.2)	68 (9.8)	0.160	235 (6.6)	42 (8.4)	0.165
Slow gait speed	1547 (33.6)	293 (42.3)	< 0.001	1126 (31.9)	168 (33.5)	0.470

The values are shown as number of cases (%) and were analyzed using the Chi-square test

*BMI* body mass index, *PF* physical fitness

**Table 5:** ORs of prevalence and factors associated with sarcopenia screened by finger-circle test on multivariate analyses

Variables [Reference]	Categories	Total ^a^		Settsu City		Hannan City	
		ORs (95% CI)	*p*-value	ORs (95% CI)	*p*-value	ORs (95% CI)	*p*-value
Age [40–49 years]	50–59	1.04 (0.82 to 1.33)	0.736	0.94 (0.68 to 1.29)	0.681	1.21 (0.83 to 1.77)	0.328
	60–69	1.41 (1.13 to 1.75)	0.003	1.43 (1.07 to 1.90)	0.014	1.37 (0.96 to 1.96)	0.081
	70–79	2.33 (1.87 to 2.90)	< 0.001	2.22 (1.68 to 2.94)	< 0.001	2.47 (1.73 to 3.50)	< 0.001
	≥80	3.23 (2.44 to 4.26)	< 0.001	2.85 (1.98 to 4.11)	< 0.001	3.90 (2.53 to 6.02)	< 0.001
Sex [Men]	Women	0.96 (0.82 to 1.13)	0.626	0.97 (0.79 to 1.19)	0.783	0.93 (0.72 to 1.20)	0.557
BMI [18.5–24.9 kg/m^2^]	< 18.5	5.80 (4.81 to 7.00)	< 0.001	4.96 (3.88 to 6.34)	< 0.001	7.26 (5.42 to 9.72)	< 0.001
	25.0–29.9	0.33 (0.26 to 0.41)	< 0.001	0.30 (0.22 to 0.40)	< 0.001	0.38 (0.27 to 0.53)	< 0.001
	≥30	0.22 (0.12 to 0.39)	< 0.001	0.17 (0.07 to 0.39)	< 0.001	0.29 (0.13 to 0.68)	0.004
Alcohol status [Non-drinker]	Drinker	0.90 (0.77 to 1.04)	0.150	0.88 (0.73 to 1.07)	0.212	0.91 (0.72 to 1.14)	0.396
Smoking status [Never-smoker]	Past	1.21 (1.02 to 1.43)	0.033	1.07 (0.85 to 1.34)	0.578	1.42 (1.08 to 1.85)	0.011
	Current	1.44 (1.18 to 1.76)	< 0.001	1.45 (1.13 to 1.86)	0.004	1.38 (1.02 to 1.93)	0.044
Living status [Living together]	Alone	0.92 (0.76 to 1.11)	0.372	0.87 (0.68 to 1.12)	0.297	1.01 (0.75 to 1.36)	0.973
Socioeconomic status [High]	Low	1.05 (0.92 to 1.20)	0.472	1.06 (0.89 to 1.27)	0.491	1.06 (0.85 to 1.31)	0.620
Self-reported health [Good]	Poor	1.45 (1.20 to 1.75)	< 0.001	1.28 (1.01 to 1.62)	0.046	1.77 (1.31 to 2.40)	< 0.001
Self-reported PF [Good]	Poor	0.90 (0.77 to 1.05)	0.186	0.93 (0.76 to 1.14)	0.473	0.86 (0.67 to 1.10)	0.229
Exercise habits per week [Yes]	No	1.03 (0.90 to 1.18)	0.674	1.05 (0.88 to 1.26)	0.573	0.99 (0.79 to 1.23)	0.898
Mastication function [Good]	Poor	1.06 (0.90 to 1.25)	0.490	1.17 (0.95 to 1.44)	0.132	0.90 (0.68 to 1.18)	0.438
Number of meals [≥3 times]	2	1.14 (0.93 to 1.39)	0.217	1.07 (0.82 to 1.39)	0.619	1.27 (0.92 to 1.75)	0.144
	1	1.82 (1.00 to 3.32)	0.049	1.80 (0.88 to 3.68)	0.110	1.83 (0.61 to 5.52)	0.280
Sleep status [Good]	Poor	1.07 (0.91 to 1.26)	0.401	1.11 (0.91 to 1.36)	0.312	1.00 (0.77 to 1.30)	0.987
Short cognitive ability [Good]	Poor	1.18 (0.94 to 1.48)	0.147	1.17 (0.87 to 1.56)	0.296	1.23 (0.85 to 1.77)	0.277
Gait speed [Normal]	Slow	1.06 (0.91 to 1.23)	0.481	1.20 (0.98 to 1.47)	0.072	0.88 (0.69 to 1.12)	0.299

The values were shown in odds ratios (95% confidence intervals)

*BMI* body mass index, *CI* confidence interval, *OR* odds ratio, *PF* physical fitness

^a^Multivariate adjusted model for the total number of participants was adjusted by adding the area (Settsu or Hannan Cities) to the covariates

## Discussion

In the present study, we determined the prevalence of sarcopenia screened by finger-circle test among community-dwelling middle-aged and older adults (age ≥ 40 years) residing in Settsu and Hannan Cities, Osaka Prefecture, Japan and conducted an exploratory analysis of factors associated with sarcopenia based on the survey items. Based on sarcopenia (defined by bioimpedance-measured SMI), we confirmed the finger-circle test as a valid method of screening sarcopenia. In all participants, age, BMI, smoking status, self-reported health, and number of meals were associated with the prevalence of sarcopenia. To the best of our knowledge, the present study is the first to determine the prevalence and factors associated with sarcopenia screened by finger-circle test in middle-aged and older Japanese adults. Our study’s findings may be useful for a population-based health approach targeting sarcopenia for prevention.

We evaluated the validity of the finger-circle test for assessing sarcopenia (defined by SMI). The finger-circle test for predicting sarcopenia (by SMI) was found to be less sensitive but more specific than reported in a previous study using calf circumference as a screening tool for sarcopenia [12]. This result implies that the finger-circle test may be likely to identify sarcopenia in individuals who do not actually have it but is highly adept at accurately indicating that sarcopenia is not present in individuals without it. It is important for screening tools to be able to cheaply and rapidly distinguish between high and low risk individuals for diseases and disorders, prior to definitive diagnostic tests in evidently healthy populations [35]. Our results indicated that the finger-circle test is sufficiently capable of screening out subjects who are less likely to have sarcopenia prior to the definitive diagnosisand therefore, is potentially useful as a screening test. A study of middle-aged and older adult Japanese found calf circumferences of 34 cm for men and 33 cm for women to be the optimal cutoff points for predicting low muscle mass (defined by SMI) [11]. Similar results were also obtained in other studies [12, 13], and the AWGS 2019 Consensus also uses these cutoff values [2]. A study of community-dwelling Japanese adults aged ≥65 years reported that the finger-circle circumference is roughly 33 and 31 cm for older adults men and women, respectively [14]. These findings showed that the cutoff values for the finger-circle and calf circumferences are relatively consistent for identifying individuals with low muscle mass (by SMI) and that the finger-circle test is a useful sarcopenia screening tool. However, the difference between the calf circumference cutoff for low muscle mass and finger-circle circumference is larger in women than in men (approximately 1 and 2 cm in men and women, respectively); these differences may affect the accuracy of the finger-circle test. In addition, the calf circumference as a screening tool for low muscle mass (defined by bioimpedance-assessed SMI) is less sensitive in women than in men (81.5% vs. 91.2) [12]. Conceivable reasons for this difference in sensitivity include the larger fat volumes in the women’s legs than in men’s legs, and edema of the lower extremities [11]. Our data also showed the tendency of lower positive predictive value in women than in men. In addition, a previous study has reported that individuals whose finger-circle test response was “smaller” had larger finger-circle circumferences than individuals with other responses [14]. These errors in finger-circle test accuracy can perhaps be partially explained by the finger-circle circumference and sex. Therefore, the use of the finger-circle test to screen for sarcopenia requires a thorough understanding of the effects of sex and finger-circle circumference on systematic error.

We found the weighted prevalence of sarcopenia screened by the finger-circle test to be 12.9% in Settsu and 12.8% in Hannan. In both cities, sarcopenia was more prevalent among the older individuals (about 16%) than among middle-aged individuals (about 9%). A meta-analysis of 109 studies of older individuals reported that the prevalence of sarcopenia, defined by the EWGSOP/AWGS criteria was 12.9% and that sarcopenia is more prevalent in oldest adults than in younger older adults [36]. In a study of community-dwelling adults in Singapore, the prevalence of sarcopenia (by the AWGS 2019 Consensus criteria) was approximately 2 and 7% in adults aged 40–49 and 50–59 years, respectively [21]. Using data from Korea National Health and Nutrition Examination Surveys, Moon et al. reported the prevalence of sarcopenia (by SMI) in adults aged 40–59 years as 3.6% [37]. From these studies, we speculated that the prevalence of sarcopenia assessed by the finger-circle test may be slightly higher in both middle-aged and older adults than the prevalence of sarcopenia defined by the gait speed, grip strength, or skeletal muscle mass. The finger-circle test is not a tool for definitively diagnosing sarcopenia but rather a screening tool that identifies individuals who are likely to have sarcopenia, which may explain why the finger-circle test yielded a higher prevalence of sarcopenia than that of the definitive diagnoses. Furthermore, skeletal muscle mass and grip strength tend to decline once individuals reach their thirties [25]; this may explain why sarcopenia also occurs in middle-aged adults. The above findings suggest that sarcopenia is not confined to the older adults and that its assessment must begin during middle age, when muscle strength and muscle mass begin to decline.

Our study showed that age, BMI, smoking status, self-reported health, and number of meals were associated with the prevalence of sarcopenia screened by finger-circle test. A cross-sectional study of community-dwelling adults aged 28–95 years found that the age of 30 years marks the start of decline not only of grip strength but also of muscle quality [38]. The lower the number of meals taken (for example, skipping breakfast), the lower the total energy intake per day [39], and this may be associated with lower BMI [40]. In addition, a meta-analysis which examined the relation between smoking and prevalence of sarcopenia found a higher prevalence among smokers than among non-smokers [22]. These intrinsic (age) and extrinsic (BMI, smoking status, and diet) factors have been identified as risk factors for sarcopenia [41], and these studies appear to support the results of our study. To the best of our knowledge, no previous study has demonstrated that self-reported health is significantly associated with the prevalence of sarcopenia [20, 21]. However, previous studies reported that among older adults, sarcopenia defined by BMI-adjusted values of skeletal muscle mass [42] and grip strength [43] is more strongly associated with the risk of disability and the risk of composite outcomes such as falls and hospitalization than sarcopenia defined by methods that do not adjust for BMI. Therefore, the AWGS 2019 Consensus states that adjusting for measured muscle mass using BMI is the best method for defining sarcopenia [2]. In our study, we showed that self-reported health was significantly associated with the prevalence of sarcopenia even after adjusting for age and BMI. One study showed that individuals with sarcopenia have a low health-related quality of life [44]. Moreover, other studies demonstrated that several self-reported diseases are similarly associated with the prevalence of sarcopenia [16, 19, 23]. However, regarding the association between self-reported health and the prevalence of sarcopenia, it is unknown whether the development of sarcopenia from the above associations causes self-reported health to decline or whether the decline of self-reported health results in sarcopenia. Although the causal relationship between self-reported health and the prevalence of sarcopenia could be in the opposite direction, these results seem to provide an important finding the possibility that poor self-reported health in community-dwelling adults needs to be resolved due to being related factors for prevalence of sarcopenia. Appropriately designed epidemiological studies are important for assessing novel risk factors that could affect healthy lifespans [45]. The prevalence and factors associated with sarcopenia in middle-aged and older adults that we demonstrated in the present study may be useful in designing policies that seek to promote health by lowering the prevalence of sarcopenia.

The strength of the present study is that it examined the prevalence of sarcopenia in two different cities in Osaka Prefecture and explored the factors associated with sarcopenia in residents who were aged ≥40 years. The results for both cities were comparable. We also confirmed that the finger-circle test is valid for screening sarcopenia in comparison to sarcopenia assessed with the actual measurement of SMI. However, the methodology of this study was limited in several ways. First, our study was cross-sectional in design. Therefore, we could not infer whether the relationship observed between prevalence of sarcopenia and its associated factors was temporal or causal. Second, finger-circle test was only moderately (not particularly strongly) predictive of the presence of sarcopenia as defined by SMI (AUROC: 0.666). In addition, to verify the validity of the finger-circle test, we assessed sarcopenia via the actual measurements, but the measurements only reflected the muscle mass. The diagnostic criteria for sarcopenia include gait speed [46] and grip strength [47], which are associated with the risk of mortality among older adults. It has been reported in some studies that sarcopenia defined only by low muscle mass was similar to sarcopenia defined according to the criteria of low muscle mass, low muscle strength, and low physical performance [28, 29]; however, other studies have shown a difference in the prevalence of sarcopenia by these criteria [6, 7]. In addition, although we used bioimpedance methods to evaluate the muscle mass, it is possibly not the most accurate analysis [1–4] to validate another tool including finger-cycle test. Therefore, the validity of the finger-circle test should be examined again and compared with sarcopenia assessment based on muscle mass as well as grip strength and gait speed [1–4]. Third, the present study was a self-administered survey, and thus, may have included systematic error due to self-reporting. Also, although we conducted an exploratory examination of many potential factors associated with sarcopenia, we could not completely consider bias associated with factors we did not measure, such as educational history [19, 48] and the presence of disease [19, 23]. Lastly, although we selected participants via stratified sampling by elementary school zone, the response rate for the mail survey was only 58.1% in Settsu and 60.0% in Hannan. Thus, these participants may have been more health-conscious than the general population, suggesting that selection bias may have been present. We found no significant difference in crude and weighted prevalence of sarcopenia screened by finger-circle test (Table 3). Therefore, we concluded that there was no serious selection bias in the sample. However, the face-to-face survey participants, among whom validity of the finger-circle test was examined, were older and included a higher percentage of women than the mail survey participants. Thus, a limitation is the ability to extrapolate the validity of the finger-circle test obtained in the face-to-face survey to the participants in the mail survey. These limitations could prevent our results from being generalized. Therefore, it is necessary to conduct prospective observational studies, and randomized controlled trials, to assess whether the factors found in the present study are in fact associated with the risk of developing sarcopenia.

Our study showed that sarcopenia may be present not only among older adults but also among middle-aged adults (ages 40–59 years). A recent paradigm shift has seen a move away from a focus on individual diseases to an awareness that several chronic diseases have shared risk factors and that multiple coexisting disease states are strongly affected by the complexity of the health trajectories, disorders, and necessary care [49]. In Japan, specific health checkups and specific health guidance have been conducted since April 2008 to assess the risk of metabolic syndrome in middle-aged and older adults aged 40–74 years [50]. To extract cases of “possible sarcopenia”, the AWGS 2019 Consensus recommends assessing the calf circumference in primary health care or community preventive services settings [2]. The finger-circle test, which does not require specialized techniques or instruments, can be performed by anyone and is a simpler sarcopenia screening tool than the assessment of the calf circumference. This aspect of the finger-circle test suggests that it should be added as a sarcopenia screening tool to the specific health checkups and specific health guidance currently conducted for middle-aged and older adults in Japan. This effort may enable the simultaneous evaluation of the risks of sarcopenia and metabolic syndrome from middle-age onward. Our study also suggested that providing individuals with an opportunity to identify their own health issue is crucial to lower the prevalence of sarcopenia screened by finger-circle test.

## Conclusions

Our study showed that the finger-circle test is a useful sarcopenia screening tool. In addition, we found that sarcopenia is present not only among older adults but also among middle-aged adults. Age, BMI, smoking status, self-reported health, and number of meals were all associated with the prevalence of sarcopenia. Our findings suggest that it is important to provide individuals with opportunities to be able to perform a test that could lead to the identification of sarcopenia, regardless of their age. The results of the present study may provide useful indications for developing public health programs, not only for the prevention, but especially for the management of sarcopenia.

## Supplementary Information

**Additional file 1: Table S1.** Comparison of residents characteristics of complete or missing case in this study. **Table S2.** Distribution of height and body composition according to finger-circle test in face to face survey. **Table S3.** Sensitivity analysis of prevalence of and factors associated with sarcopenia using complete case data.

## Data Availability

Researchers can request our study group for permission to use this data by contacting T.Y. (t-yoshida@nibiohn.go.jp) or M.M. (miyachi@nibiohn.go.jp).

## Abbreviations

AWGS: Asian Working Group for Sarcopenia
ALM: Appendicular skeletal muscle mass
AUROC: Area under the receiver operating characteristic curves
BMI: Body mass index
CI: Confidence interval
EWGSOP: European Working Group on Sarcopenia in Older People
MICE: Multivariate imputation by chained eq.
OR: Odds ratio
SMI: Skeletal muscle mass index
