# Supplementary material for: Factors associated with sarcopenia screened by finger-circle test among middle-aged and older adults: a population-based multisite cross-sectional survey in Japan
Source: BMC Public Health. 2021 Apr 26;21:798. doi: 10.1186/s12889-021-10844-3 (PMC8074487; doi:10.1186/s12889-021-10844-3)
Supplement: Supplementary file 1 — Additional file 1: Table S1. Comparison of residents characteristics of complete or missing case in this study. Table S2. Distribution of height and body composition according to finger-circle test in face to face survey. Table S3. Sensitivity analysis of prevalence of and factors associated with sarcopenia using complete case data. [file 12889_2021_10844_MOESM1_ESM.docx]

**Supporting information**

Factors associated with sarcopenia screened by finger-circle test among middle-aged and older adults: a population-based multisite cross-sectional survey in Japan

Daiki Watanabe, Tsukasa Yoshida, Takashi Nakagata, Naomi Sawada, Yosuke Yamada, Kayo Kurotani, Kenji Tanaka, Megumi Okabayashi, Hidekazu Shimada, Hidemi Takimoto, Nobuo Nishi, Keiichi Abe, and Motohiko Miyachi

**Table S1** Comparison of residents characteristics of complete or missing case in this study

**Table S2** Distribution of height and body composition according to finger-circle test in face to face survey

**Table S3** Sensitivity analysis of prevalence of and factors associated with sarcopenia using complete case data

Table S1 Comparison of residents characteristics of complete or missing case in this study

|  | Settsu City | | | | *p*-value |  | Hannan City | | | | *p*-value |
| --- | --- | --- | --- | --- | --- | --- | --- | --- | --- | --- | --- |
|  | Complete case  (*n* = 4722) | | Incomplete case (*n* = 578) | |  |  | Complete case (*n* = 3576) | | Incomplete case (*n* = 461) | |  |
|  | *n* | (%) | *n* | (%) |  |  | *n* | (%) | *n* | (%) |  |
| Age [years] |  |  |  |  |  |  |  |  |  |  |  |
| 40-49 | 1000 | (21.2) | 104 | (18.0) | <0.001 |  | 624 | (17.4) | 77 | (16.7) | <0.001 |
| 50-59 | 892 | (18.9) | 73 | (12.6) |  |  | 714 | (20.0) | 71 | (15.4) |  |
| 60-69 | 1225 | (25.9) | 118 | (20.4) |  |  | 989 | (27.7) | 99 | (21.5) |  |
| 70-79 | 1296 | (27.4) | 192 | (33.2) |  |  | 999 | (27.9) | 145 | (31.5) |  |
| ≥80 | 309 | (6.5) | 91 | (15.7) |  |  | 250 | (7.0) | 69 | (15.0) |  |
| Women | 2238 | (47.4) | 258 | (44.6) | 0.210 |  | 1750 | (48.9) | 215 | (46.6) | 0.353 |
| Sarcopenia | 606 | (12.8) | 86 | (14.9) | 0.168 |  | 438 | (12.2) | 64 | (13.9) | 0.317 |

The values are shown as number of cases (%) and were analyzed using the Chi-square test.

Table S2 Distribution of height and body composition according to finger-circle test in face to face survey

|  | Finger-circle test ^a^ | | | | | | *p* for trend ^b^ |
| --- | --- | --- | --- | --- | --- | --- | --- |
|  | Bigger | | Same size | | Smaller | |  |
| **Total (*n* = 525)** | 234 | | 215 | | 76 | |  |
| Height [cm] | 154.3 | (7.5) | 154.2 | (7.8) | 154.5 | (7.3) | 0.892 |
| Body weight [kg] | 57.4 | (9.7) | 51.6 | (7.0) | 48.0 | (8.5) | <0.001 |
| BMI [kg/m^2^] | 24.1 | (3.2) | 21.7 | (2.3) | 20.1 | (2.9) | <0.001 |
| LBM [kg] | 38.8 | (6.7) | 36.7 | (5.5) | 35.6 | (5.6) | <0.001 |
| Body fat [kg] | 18.6 | (6.8) | 14.9 | (4.5) | 12.3 | (5.4) | <0.001 |
| ALM [kg] | 16.5 | (3.4) | 15.6 | (2.9) | 15.2 | (2.9) | <0.001 |
| SMI [kg/m^2^] | 6.9 | (0.8) | 6.5 | (0.6) | 6.3 | (0.7) | <0.001 |
| **Women (*n* = 436)** | 189 | | 182 | | 65 | |  |
| Height [cm] | 152.0 | (5.7) | 152.0 | (5.8) | 152.8 | (6.1) | 0.478 |
| Body weight [kg] | 55.6 | (9.0) | 50.0 | (5.9) | 46.4 | (7.2) | <0.001 |
| BMI [kg/m^2^] | 24.0 | (3.4) | 21.7 | (2.4) | 19.9 | (2.8) | <0.001 |
| LBM [kg] | 36.1 | (3.4) | 34.7 | (2.7) | 33.9 | (3.1) | <0.001 |
| Body fat [kg] | 19.5 | (6.9) | 15.3 | (4.5) | 12.5 | (5.3) | <0.001 |
| ALM [kg] | 15.3 | (2.1) | 14.6 | (1.7) | 14.4 | (1.9) | <0.001 |
| SMI [kg/m^2^] | 6.6 | (0.5) | 6.3 | (0.4) | 6.1 | (0.5) | <0.001 |
| **Men (*n* = 89)** | 45 | | 33 | | 11 | |  |
| Height [cm] | 163.8 | (6.6) | 166.5 | (5.4) | 164.7 | (5.9) | 0.239 |
| Body weight [kg] | 65.1 | (8.4) | 60.1 | (6.4) | 57.6 | (9.8) | <0.001 |
| BMI [kg/m^2^] | 24.2 | (2.5) | 21.7 | (1.8) | 21.2 | (3.1) | <0.001 |
| LBM [kg] | 50.0 | (5.3) | 47.5 | (4.2) | 46.0 | (5.9) | 0.005 |
| Body fat [kg] | 15.1 | (5.0) | 12.6 | (4.0) | 11.6 | (6.4) | 0.009 |
| ALM [kg] | 21.5 | (3.5) | 20.6 | (2.8) | 19.9 | (3.5) | 0.083 |
| SMI [kg/m^2^] | 8.0 | (0.8) | 7.4 | (0.7) | 7.3 | (1.0) | 0.002 |
| **<65 years (*n* = 107)** | 53 | | 46 | | 8 | |  |
| Height [cm] | 159.0 | (8.0) | 158.0 | (7.2) | 155.4 | (8.4) | 0.245 |
| Body weight [kg] | 61.9 | (12.7) | 52.6 | (6.2) | 45.0 | (5.9) | <0.001 |
| BMI [kg/m^2^] | 24.4 | (4.4) | 21.0 | (1.9) | 18.6 | (1.8) | <0.001 |
| LBM [kg] | 41.7 | (7.9) | 37.8 | (5.4) | 34.0 | (3.8) | <0.001 |
| Body fat [kg] | 20.2 | (9.4) | 14.8 | (3.9) | 11.1 | (2.7) | <0.001 |
| ALM [kg] | 18.5 | (4.1) | 16.5 | (2.9) | 14.8 | (2.2) | <0.001 |
| SMI [kg/m^2^] | 7.2 | (1.0) | 6.5 | (0.6) | 6.1 | (0.5) | <0.001 |
| **≥65 years (*n* = 418)** | 181 | | 169 | | 68 | |  |
| Height [cm] | 152.9 | (6.8) | 153.2 | (7.6) | 154.4 | (7.3) | 0.191 |
| Body weight [kg] | 56.1 | (8.1) | 51.3 | (7.1) | 48.3 | (8.8) | <0.001 |
| BMI [kg/m^2^] | 24.0 | (2.8) | 21.8 | (2.4) | 20.2 | (3.0) | <0.001 |
| LBM [kg] | 37.9 | (6.0) | 36.4 | (5.5) | 35.8 | (5.7) | 0.004 |
| Body fat [kg] | 18.2 | (5.8) | 14.9 | (4.7) | 12.5 | (5.7) | <0.001 |
| ALM [kg] | 16.0 | (3.0) | 15.3 | (2.8) | 15.2 | (3.0) | 0.024 |
| SMI [kg/m^2^] | 6.8 | (0.7) | 6.5 | (0.6) | 6.3 | (0.8) | <0.001 |

ALM, appendicular skeletal muscle mass; BMI, body mass index; LBM, lean body mass; SMI, skeletal muscle mass index

^a^ All variables were shown in mean (standard deviation).

^b^ *p*-value of linear trends was calculated using the exposure variable of finger-circle test as a continuous variable.

^c^ Skeletal muscle mass index (SMI) was calculated as the appendicular skeletal muscle mass (kg) divided by the square of the height (m).

Table S3 Sensitivity analysis of prevalence of and factors associated with sarcopenia using complete case data

| Variables [Reference] | Categories | Total ^a^ | | |  | Settsu City | | |  | Hannan City | | |
| --- | --- | --- | --- | --- | --- | --- | --- | --- | --- | --- | --- | --- |
|  |  | ORs (95%CI) | | *p*-value |  | ORs (95%CI) | | *p*-value |  | ORs (95%CI) | | *p*-value |
| Age [40-49 years] | 50-59 | 1.15 | (0.89 to 1.48) | 0.281 |  | 0.94 | (0.75 to 1.47) | 0.772 |  | 1.28 | (0.86 to 1.90) | 0.223 |
|  | 60-69 | 1.48 | (1.17 to 1.88) | 0.001 |  | 1.43 | (1.16 to 2.12) | 0.004 |  | 1.35 | (0.93 to 1.97) | 0.114 |
|  | 70-79 | 2.47 | (1.96 to 3.12) | <0.001 |  | 2.22 | (1.85 to 3.37) | <0.001 |  | 2.39 | (1.65 to 3.48) | <0.001 |
|  | ≥80 | 3.34 | (2.46 to 4.55) | <0.001 |  | 2.85 | (2.17 to 4.88) | <0.001 |  | 3.49 | (2.17 to 5.62) | <0.001 |
| Sex [Men] | Women | 1.00 | (0.84 to 1.18) | 0.975 |  | 0.98 | (0.79 to 1.22) | 0.838 |  | 1.01 | (0.77 to 1.33) | 0.921 |
| BMI [18.5-24.9 kg/m^2^] | <18.5 | 5.67 | (4.65 to 6.90) | <0.001 |  | 4.93 | (3.81 to 6.39) | <0.001 |  | 6.95 | (5.11 to 9.44) | <0.001 |
|  | 25.0-29.9 | 0.33 | (0.26 to 0.41) | <0.001 |  | 0.31 | (0.23 to 0.42) | <0.001 |  | 0.35 | (0.24 to 0.51) | <0.001 |
|  | ≥30 | 0.26 | (0.14 to 0.46) | <0.001 |  | 0.20 | (0.09 to 0.46) | <0.001 |  | 0.35 | (0.15 to 0.80) | 0.013 |
| Alcohol status [Non-drinker] | Drinker | 0.92 | (0.79 to 1.08) | 0.325 |  | 0.91 | (0.74 to 1.13) | 0.401 |  | 0.93 | (0.73 to 1.19) | 0.556 |
| Smoking status [Never-smoker] | Past | 1.22 | (1.01 to 1.46) | 0.037 |  | 1.13 | (0.88 to 1.43) | 0.338 |  | 1.34 | (1.01 to 1.78) | 0.045 |
|  | Current | 1.49 | (1.21 to 1.85) | <0.001 |  | 1.54 | (1.18 to 2.01) | 0.001 |  | 1.36 | (1.02 to 1.79) | 0.044 |
| Living status [Living together] | Alone | 0.91 | (0.74 to 1.12) | 0.355 |  | 0.83 | (0.63 to 1.09) | 0.184 |  | 1.05 | (0.76 to 1.45) | 0.772 |
| Socioeconomic status [High] | Low | 1.03 | (0.89 to 1.20) | 0.660 |  | 1.07 | (0.88 to 1.29) | 0.504 |  | 1.00 | (0.80 to 1.26) | 0.999 |
| Self-reported health [Good] | Poor | 1.46 | (1.19 to 1.79) | <0.001 |  | 1.33 | (1.03 to 1.72) | 0.029 |  | 1.69 | (1.21 to 2.34) | 0.002 |
| Self-reported PF [Good] | Poor | 0.90 | (0.76 to 1.07) | 0.229 |  | 0.92 | (0.74 to 1.14) | 0.438 |  | 0.88 | (0.67 to 1.15) | 0.344 |
| Exercise habits per week [Yes] | No | 1.04 | (0.90 to 1.21) | 0.575 |  | 1.09 | (0.90 to 1.33) | 0.358 |  | 0.97 | (0.77 to 1.22) | 0.790 |
| Mastication function [Good] | Poor | 1.05 | (0.88 to 1.25) | 0.619 |  | 1.11 | (0.89 to 1.39) | 0.369 |  | 0.96 | (0.72 to 1.29) | 0.802 |
| Number of meals [≥3 times] | 2 | 1.16 | (0.94 to 1.44) | 0.177 |  | 1.13 | (0.85 to 1.49) | 0.394 |  | 1.23 | (0.88 to 1.73) | 0.229 |
|  | 1 | 1.96 | (1.05 to 3.65) | 0.034 |  | 2.00 | (0.97 to 4.15) | 0.061 |  | 1.77 | (0.52 to 5.98) | 0.359 |
| Sleep status [Good] | Poor | 1.05 | (0.89 to 1.25) | 0.550 |  | 1.12 | (0.90 to 1.39) | 0.320 |  | 0.96 | (0.73 to 1.28) | 0.796 |
| Short cognitive ability [Good] | Poor | 1.15 | (0.90 to 1.47) | 0.272 |  | 1.17 | (0.86 to 1.59) | 0.318 |  | 1.11 | (0.74 to 1.66) | 0.626 |
| Gait speed [Normal] | Slow | 1.02 | (0.86 to 1.20) | 0.837 |  | 1.14 | (0.92 to 1.42) | 0.219 |  | 0.85 | (0.65 to 1.11) | 0.242 |

BMI, body mass index; CI, confidence interval; OR, odds ratio; PF, physical fitness

The values were shown in odds ratios (95% confidence intervals).

^a^ Multivariate adjusted model for total participants was adjusted by adding area (Settsu or Hannan Cities) in covariate variables.
